# Dendrimers: A New Race of Pharmaceutical Nanocarriers

**DOI:** 10.1155/2021/8844030

**Published:** 2021-02-15

**Authors:** Pooja Mittal, Anjali Saharan, Ravinder Verma, Farag M. A. Altalbawy, Mohammed A. Alfaidi, Gaber El-Saber Batiha, Wahida Akter, Rupesh K. Gautam, Md. Sahab Uddin, Md. Sohanur Rahman

**Affiliations:** ^1^MM School of Pharmacy, Maharishi Markandeshwar University, Sadopur, Ambala, Haryana 134007, India; ^2^Maharishi Dayanand University, Rohtak, Haryana 124001, India; ^3^Department of Biological Sciences, University College of Duba, Tabuk University, Duba 71911, Saudi Arabia; ^4^National Institute of Laser Enhanced Sciences (NILES), Cairo University, Giza 12613, Egypt; ^5^Department of Pharmacology and Therapeutics, Faculty of Veterinary Medicine, Damanhour University, Damanhour 22511, Al Beheira, Egypt; ^6^Department of Pharmaceutical Sciences, North South University, Dhaka 1229, Bangladesh; ^7^Department of Pharmacy, Southeast University, Dhaka, Bangladesh; ^8^Pharmakon Neuroscience Research Network, Dhaka, Bangladesh; ^9^Department of Biochemistry and Molecular Biology, Trust University, Barishal, Ruiya, Nobogram Road, Barishal 8200, Bangladesh

## Abstract

Dendrimers are nanosized, symmetrical molecules in which a small atom or group of atoms is surrounded by the symmetric branches known as dendrons. The structure of dendrimers possesses the greatest impact on their physical and chemical properties. They grow outwards from the core-shell which further reacts with monomers having one reactive or two dormant molecules. Dendrimers' unique characteristics such as hyperbranching, well-defined spherical structure, and high compatibility with the biological systems are responsible for their wide range of applications including medical and biomedical areas. Particularly, the dendrimers' three-dimensional structure can incorporate a wide variety of drugs to form biologically active drug conjugates. In this review, we focus on the synthesis, mechanism of drug encapsulations in dendrimers, and their wide applications in drug delivery.

## 1. Introduction

Drug delivery is the utmost part of any dosage unit. In order to ensure the targeted and effective drug delivery on an appropriate site without any configuration changes, preventing degradation, therapeutic activity, and stability, various polypeptide molecules are used. Among which, one used as nanocarriers is called dendrimers [1]. The word dendrimer is coined by a Greek term known as dendron which means “branching of a tree.” Dendrimers are polymeric globular branched and symmetrical structures with defined shape and specificity. Dendrimers are highly defined nanoparticles with sizes varying from 1 to 15 nanometer (nm). Dendrimers are primarily used to enhance the specific property of a compound [2, 3]. By using the dendritic method, enhancement of drug activity is achieved by applying different dendritic patterns through which the functionality of a certain functional compound can enhance greatly the sum of single entries placed on the surface [4]. This enhanced activity is due to the synergistic effect achieved by dendrites. This configured nature leads to evolution in the field of dendritic polymer formulations.

Dendrimers are primarily used to achieve some specific goals/targets such as to modify and enhance the bioavailability of drugs by altering the pharmacokinetic and pharmacodynamic properties of the active moiety [5]. The active moiety of the drug molecule has a significant role in drug delivery by promoting controlled and targeted drug delivery to a specific site achieved by reducing the size of the drug. Optical properties are also exhibited by them such as fluorescence, which helps in the determination of particle diameter and size [6].

### 1.1. Applications of Dendrimers

Dendrimers are classified broadly into two categories as medical and nonmedical usage.

#### 1.1.1. Medical Applications

*(1) Biomedical Study*. Dendrimers are widely used in the biomedical field where they are used as analogs to proteins, enzymes, and viruses where they are primarily used to focus the target cells and conjugated to the host dendrimeric cells, for example, poly(amidoamine) dendrimer [7].

*(2) Magnetic Resonance*. Dendrimers are extensively used in magnetic resonance to improve the contrasts of the image. For example, metallic dendrimers are used to create the magnetic resonance imaging contrast agent [8].

*(3) Biomimics*. Dendrimers are also used to mimic the variety of biomolecules and create the microenvironment [9].

#### 1.1.2. Solubility Enhancement

Dendrimers help in improving the solubility profile of the poor and sparingly soluble drugs which results in increased bioavailability of drugs [10].

#### 1.1.3. Stability Enhancement

Dendritic formulation aids in the stability of the ingredients inside the core and provides dynamic internal cavities where neutral molecules and ions can be placed to prevent degradation.

#### 1.1.4. Targeted Delivery

Dendrimers also aid in site-specific targeted drug delivery via targeting ligands and conjugate to the dendrimer surface [11].

#### 1.1.5. Dummy and Carrier for Formulations

Dendrimer molecules have the ability to cross the cell membranes because of uniform size; due to this property, they help in various pharmacological activities [12].

#### 1.1.6. Nanoparticles

Poly(amidoamine) (PAMAM) dendrimers are used as a nanoparticle because it has a tertiary amine group at the branching point. Metal ions are introduced in the aqueous solution of dendrimers, and metal ions form a complex with the lone pair of electrons present at the tertiary amines. The ions are then reduced to the zero-valent state to form the nanoparticles that are encapsulated within the dendrimer [11].

#### 1.1.7. Nanodrugs

Various dendrimeric formulations were used as nanodrugs in various diseases such as polylysine (PPL) dendrimers with sulfonated naphthyl groups which were used as antivirus. PPL dendrimers with tertiary alkylammonium groups attached to the surface are antibacterial, and chitosan dendrimer hybrids are used as antibacterial agents [6].

#### 1.1.8. Hydrogel for Ocular Drug Delivery

Dendrimeric formulations which are used in the hydrogels are cross-linked networks that increase the volume in the aqueous solution. By the addition of polyethylene glycol groups, they are widely used in cartilage tissue production and for sealing the ophthalmic injuries and targeted delivery [7].

#### 1.1.9. Transdermal Drug Delivery

Dendrimers are found to enhance solubility and plasma circulation via transdermal formulation. PAMAM dendrimers make complexes with the nonsteroidal anti-inflammatory drugs and lead to enhanced permeation through the skin and act as permeation enhancers, for example, indomethacin [11].

All these resulting benefits lead to an exponential increase in the rapid and efficient synthesis of dendrimers along with their numerous applications in various areas such as catalysis, electronics, sensing, nanoengineering, diagnostics, and drug and gene delivery. Some examples of dendrimers that exhibited these properties are PAMAM, poly(propylene imine) (PPI), poly(L-lysine) (PLL), and triazene-based dendrimers [13].

**Figure 1:**
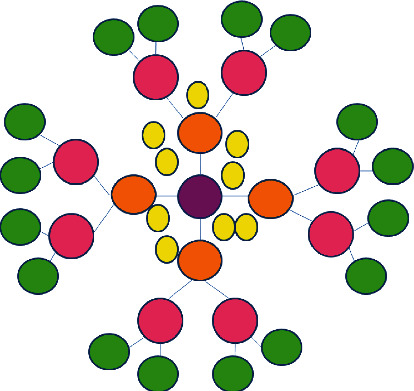
Basic structure of dendrimer.

The dendrimers' role in drug delivery is the utmost part of any dosage form to achieve its biopharmaceutical and pharmacological activity and benefits. Dendrimers act in two manners for drug delivery as in the formulation and nanoconstruct [14]. Drugs are entrapped using noncovalent interactions whereas in nanoconstruct, dendrimers are covalently bonded. The phenomenon was explained using the use of PAMAM dendrimers [15]. The structure having cavities along with an abundant terminal group results in spherical and defined branching of polymers resulting in the formation of stable complexes with drugs such as plasmid DNA, oligonucleotides, and antibodies. As reported, amine-terminated PAMAM dendrimers can solubilize different hydrophobic groups belonging to different families because the cationic charge present on the surface of the molecules disturbs the functionality of the cell membrane [16]. These modification changes lead to changes such as becoming more sensitive, effective increase of transdermal permeation, and specific drug targeting. Some examples of surface modifications are using PEGylation, acetylation, glycosylation, and amino acid functionalization leading to changes in the peripheral amine groups by neutralization which helps to improve dendrimer biocompatibility [17]. Dendritic platforms can also be used to create nanodevices by altering and modifying the binding of ligands and imaging molecules. Dendrimer nanotechnology is in the current line issue due to its multifunctional ability to produce next-generation devices. The basic structure of dendrimers is given in Figure 1.

### 1.2. Chemical Components of Dendrimers

Dendrimer moiety consists of mainly a central core atom, secondly repetitive branching units, and terminal groups which affect the functionality of molecules. An increase in the generation of branching leads to the formation of different globular structures [18]. Drug and oligo nucleic acids encapsulate in internal cavities bounded via hydrophobic and electrostatic interactions [4]. The two strategies which are used for dendrimer synthesis are mainly known as divergent and convergent [19]. As in the divergent method of synthesis, firstly coined by Tomalia, a central reactive core and reactive core were used in the growth of successive generations by only altering the peripheral molecules [20]. In successive generations, it was observed that the new dendrimers formed have molar mass doubled successively with each group resulting in the production of a large number of dendrimers.

In the convergent method, synthesis was firstly coined by Hawker and Fréchet which was defined as dendrimers containing a multifunctional core which reacts with several dendrons resulting in the fixation of dendrons resulting in a final hyperbranched product [21]. The prime advantage of this method is that dendrimers formed using this method are simple and the purification of the final reaction product has the precise placement of functional groups at the periphery with minimum defects [8].

Apart from the two synthesis reactions of dendrimers, the synthetic methods are also used due to their wide range of acceptability as they have precise control of their size, shape, number of end groups, and surface functionalities [22]. On the basis of this, a variety of dendrimers were discovered which are compositionally different resulting in a variety of chemical surface modifications as mentioned in the literature. Different types of Starburst® dendrimers have been synthesized in the last few decades such as PAMAM which is the first dendrimer family to be commercialized [23]. These dendrimers were synthesized by a divergent method initiating from an ethylene diamine or amine core which is commercially available with a diameter of 15 nm.

### 1.3. Synthesis of Dendrimers

Dendrimer synthesis lies between polymer and molecular chemistry. As they resemble molecular chemistry due to the linkage of step to step synthesis and polymer synthesis due to repetitive structure monomers [24], the route of synthesis of dendrimers varies from the divergent or convergent approach. As in the divergent method, the synthesis originates from the central moiety along with substitution branches attached to it in a significant manner whereas in the convergent method, the synthesis started from the exterior part of the molecule along with the substitution of outermost branches which results in the formation of a new dendrimer molecule [25]. In this type of synthesis method, the basic structure of the output molecule is predefined and determined by using the counts of the branches associated with it [2]. Then, the new periphery of the molecule is activated for various reactions with monomers [26].

**Figure 2:**
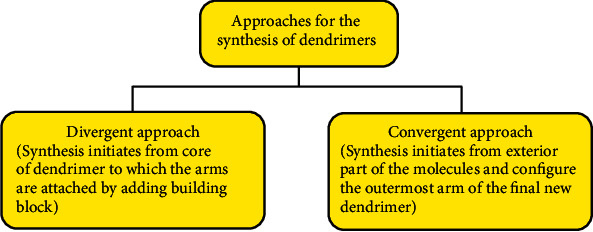
Approaches for the synthesis of dendrimers.

The foremost base and foundation of these syntheses are cascade reactions. These are basic and iterative methods currently used for synthesis. These reactions are mainly used in solid-phase peptide synthesis and in biochemical pathways among which one is fatty acid biosynthesis [17]. The two approaches and methods for the synthesis of dendrimers are mentioned in Figure 2.

### 1.4. Properties of Dendrimers

The linear polymers are prepared by classical polymerization processes which are usually random in nature and of various sizes, but their size and molecular mass can be managed using the controlled amide synthesis which creates the formation of their molecular structure. As a result of which, they were found to have enhanced chemical and physical features as compared to linear polymers [27].

The properties of different dendrimers such as solubility, chemical reactivity, and glass transition temperature depend on the nature of the end group. Dendrimer solubility also varies with the change in the nature of functional groups. The presence of hydrophilic groups makes high solubility in polar solvents whereas hydrophobic groups make their presence in nonpolar solvents. Their increase in generation leads to increased volume cubically. Some properties of dendrimer are shown in Table 1.

## 2. Classification of Dendrimers on the basis of Property

Dendrimers vary from each other in their physical and chemical properties, though they have a similar geometric architecture. The chemical properties of the branching element (dendrons) and surface groups solely decide their physical nature. The different families of dendrimers are discussed below:

### 2.1. Hydrophilic Dendrimers

They are characterized as the foremost synthesized and marketed PAMAM dendrimers. The starting reaction involves the Michael addition reaction which takes place in between an alkyl diamine core (ethylenediamine) using monomers of methyl acrylate which results in the production of branched intermediate. These newly formed monomers were further converted into small generation molecules such as −OH and −NH surface group moieties formed upon reaction with ethanolamine and excess ethylenediamine, respectively. This intermediate liberates the smallest anionic dendrimers with four −COOH groups on hydrolysis of methyl ester. The dendrimer growth reaches a critical point, and a decrease in synthetic yield is observed. This phenomenon is due to the stearic factor which is the result of overcrowding of branching arms. This method was coined as a dense packing effect. They are also considered suitable carriers for the delivery of drug molecules due to their greater aqueous solubility, large variety of surface groups, and unique structure. They are commercially available as methanol solutions are their marketed products. Starburst® dendrimers is a trademark name for its subclass which contains a tris-aminoethylene-imine core [28].

Fréchet-type dendrimers are the more recent type of dendrimer which have −COOH groups (act as a good anchoring point and increase its solubility) and developed by Hawkerand Fréchet [29]. Radially layered poly(amidoamine organosilicon) (PAMAMOS) dendrimer is the first marketed silicon-containing dendrimers that are also known as PAMAMOS. They consist of hydrophilic, nucleophilic PAMAM interiors and hydrophobic organosilicon (OS) exteriors. SARSOX is a commercially available PAMAMOS. PPI generally contains end groups of polyalkyl amines and several tertiary trispropylene amines present in the core, and these are available up to G5 and commercially available as astromol.

### 2.2. Biodegradable Dendrimers

The emergence of biodegradable dendrimers was to generate desired large molecular weight polymers that can attain a high deposition in tissue and allow a fast elimination of its fragments through urine to avoid nonspecific toxicity. These are generally formulated by the inclusion of ester groups by chemical and/or enzymatic cleavage in physiological solutions. The controlling factors include the nature of chemical bonds, the lipophilicity of monomeric units, size of dendrimers, and cleavage susceptibility of the peripheral and internal structures of dendrimers. Polyester dendrimers are used for anticancer and gene therapy because of their biodegradability and biocompatibility. However, the nonspecific hydrolysis mechanism and long-term degradation have switched to further research for obtaining specific spatial and temporal degradation behavior [30].

### 2.3. Amino Acid-Based Dendrimers

Amino acid (AA) dendrimers were formed using the integration of blocks which are having different properties such as chirality, hydrophobicity, biorecognition, and optical property. The chirality in the atom was formed due to the combined effect of the core and branching unit molecules with surface ending groups. The specific internal composition originated by AA building blocks provides stereoselective sites, where guest molecules can be attached noncovalently. These dendrimers also can be used as protein mimetic, gene, and targeted drug delivery due to their unique structural folding of the branching units. These families of dendrimers are generally synthesized either from AAs or peptide grafting and displayed in the traditional dendrimer surface or attachment of AA or peptides to a peptide or organic core [30].

### 2.4. Glycodendrimers

The origin of glycol dendrimers is based on the fact that the carbohydrate interacts with various receptors shown on the cell surface, which in turn controls several normal and abnormal processes. This interaction was found to be strong for a multivalent ligand-receptor system. It was concluded from the various studies that carbohydrates were used as the carrier in dendrimers. Glycodendrimers were reported to be utilized as a carrier for cancer therapy, as a metastatic agent, and as an immune stimulant [30].

### 2.5. Hydrophobic Dendrimers

The systemic delivery of dendrimers requires sufficient aqueous solubility. But the hydrophobic void areas in the dendritic structure facilitate the superior encapsulation and solubilization of lipophilic moieties. This structure mimics the amphiphilic polymer micelle, but not having a critical micellar concentration (CMC). The building units of dendrimers are covalently attached to each other and resist breaking down in the dilute solution phase. Dendrimers having hydrophobic internal voids and hydrophilic surfaces resembling unimolecular micelle have been reported, and the solubility of hydrophobic probes, dyes, and fluorescent markers has been studied successfully. Cyclophanes or dendrophanes are dendrimers reported to encapsulate aliphatic and aromatic moieties. These kinds of dendritic structures were also reported to control the release of drugs [30].

### 2.6. Asymmetric Dendrimers

Gillies and Fréchet [31] synthesized the most recognized asymmetrical dendrimers which are called bow tie polyester dendrimers that may offer a better pharmacokinetic profile. These are generally synthesized by coupling dendrons of various generations to a linear core molecule. The final structure forms a nonuniform orthogonal dendritic architecture. The molecular weight, structure, and number of functional groups can be tuned in this type of dendrimers. Lee and coworkers [32] utilized click chemistry for synthesizing a G3 asymmetric dendrimer.

## 3. Classification of Dendrimers on the basis of Structure

The shape, structure, branching, solubility, chirality, and attachment of dendrimer's types are discussed below:

### 3.1. Simple Dendrimers

These types of dendrimers consist of simple monomeric units which are based upon symmetrical substitution of benzene tricarboxylic acid ester. They have 4, 10, 22, and 46 benzene rings linked symmetrically and molecular diameters of 45 Å [29, 30].

### 3.2. Crystalline Dendrimers

These types of dendrimers are formed by mesogenic monomers which are produced by the functionalization of carbosylane [33].

### 3.3. Chiral Dendrimers

In these types of dendrimers, the chirality depends on the building of 4 constitutionally different but chemically similar branches to a chiral core, for example, chiral dendrimers obtained from pentaerythritol [34].

### 3.4. Micellar Dendrimers

These types of dendrimers are fully aromatic, water-soluble hyperbranched polypropylene dendrimers generating a cluster of aromatic polymeric chain that is capable to create a milieu that resembles some micellar structures which results in complex with small organic molecules in water [34, 35].

### 3.5. Hybrid Dendrimers

These dendrimers are formed by the changes in the functionalization of peripheral amines of zero generation polyethyleneimine which results in the formation of structural diverse columnar and cubic-like organized structures which were significantly transformed to produce dendritic structures, for example, hybrid dendritic linear polymers [35].

### 3.6. Amphiphilic Dendrimers

Amphiphilic dendrimers are mainly prepared by the segregation of the two sides of the chain with one having electron-withdrawing and the other part electron-donating, for example, superfect, hydraamphiphiles, and bolaamphiphiles [27, 35].

### 3.7. Metallodendrimers

Metallodendrimers are formed by a complex formation method which takes place either at the peripheral surface or in the interior of the molecule. The dendrimers formed by this method were found to possess both electrochemical and luminescence properties, for example, ruthenium bipyridine [29].

### 3.8. Tectodendrimers

Stratus® CS Acute Care™ and Starburst® are commercially available tectodendrimers. They contain dendrimer in the core and play various roles ranging from identification of diseased cell to diagnosis of infection condition [29].

### 3.9. Multilingual Dendrimers

VivaGel is a commercially available multilingual dendrimer. This contains multiple copies of a specific group of functions on the surface [34].

### 3.10. Multiple Antigen Peptide Dendrimers

Multiple antigen peptide (MAP) dendrimers have a dendron-like structure formed using the poylysineskelton. Lysine helps in the conjugation of the alkyloamine side chain which is a monomer for the various branching units. These types of dendrimers were formed and found to have numerous biological applications such as in vaccine formation and for diagnostic purposes [36].

## 4. Modes of Drug Encapsulation in Dendrimers

The phenomenon of the release of the drug depends on the type of dendrimer and core moiety used. The drug release pattern follows different mechanisms such as physical encapsulation, electrostatic encapsulation, and covalent conjugation.

### 4.1. Physical Encapsulation

In this method, the guest molecules were entrapped in the inner moiety of the macromolecule due to changes in their shape, cavities, and structural designs. The internal cavities remain vacant having two groups such as lipophilic and hydrophobic interactions, which cause an interaction with the medicament molecules of nitrogen or oxygen atoms along with the release of the hydrogen bond. The hydrogen bonding took place via several interactions such as physical and hydrogen bonding. These are suitable for the solubilization of a variety of medicaments such as anticancer drugs like doxorubicin hydrochloride and methotrexate [37].

### 4.2. Electrostatic Interactions

In this method of encapsulation, the interaction takes place from the surface of dendrimers as they contain a large number of −NH_2_ groups and −COOH groups which are used for enhancing the solubility of lipophilic medicaments. The easily ionizable drug forms complexes with the multifunctional surfaces of dendrimers having terminal groups such as ibuprofen, ketoprofen, diflunisal, naproxen, and indomethacin [37].

### 4.3. Covalent Conjugation

This method of conjugation is used for the compound having functional groups present on their outer surface. In this method, the conjugation takes place via chemical and enzymatic breakdown of hydrophilic labile bonds. Apart from it, the drugs can also be conjugately bonded through some spacer such as polyethylene glycol p-aminobenzoic acid, p-aminohippuric acid, and lauryl chains; with the use of a spacer, the drug's stability and kinetics get enhanced, for example, penicillin V, venlafaxine, 5-aminosalicylic acid, naproxen, and propranolol conjugated with PAMAM dendrimers. As a result of which, the solubility and controlled discharge of medicaments get increased [38]. Some of the common examples are given in Table 2.

## 5. Dendrimers as Drug Delivery Agents

Over the past 30 years, great attention was given to developing sustained release drug delivery systems, and the polymeric drug delivery systems are most focused among all the systems. Dendrimers or dendritic polymers have a well-defined nanosized structure which makes them appropriate for oral, parenteral, pulmonary, and nasal drug delivery. Both hydrophilic and lipophilic drugs can be delivered by dendrimers. They had shown massive potential as a drug delivery carrier because they can cross the cell membrane by both transcellular and paracellular pathways. The number and ratio of dendrimer surface groups can be modified, and hence, the related parameters like biodistribution, receptor-mediated targeting, and release rate from the dendrimers are also modifiable. Dendrimers were found to be utilized as drug delivery agents for the treatment of various diseases like HIV infection and herpes simplex virus infection, as anti-inflammatory agents, as antidotes, and as anti-Alzheimer's, anticoagulants, and anticancer agents. One formulation, i.e., VivaGel, produced by Starpharma Holdings Limited has completed clinical phase I trials and is under phase II trials which are made for the treatment of herpes simplex virus [44]. The number of diseases can be benefitted therapeutically by the application of dendrimers, but cancer treatment and detection is one area which has drawn the attention of most researchers. Dendrimers can deliver the drugs via all routes, viz., oral, intranasal, intramuscular, and intravenous routes. Drug delivery via various routes by using dendrimers as drug delivery agents is explained in the following sections [27, 30, 32].

### 5.1. Oral Drug Delivery System

As stated earlier, dendrimers are the branched tree-shaped repeating units of molecules containing a therapeutic entity inside the cavity. This property of dendrimers can be exploited to treat severe diseases like cancer. The different polymers have different branching capacities depending on their molecular weight and structure, and then accordingly, they have different drug payload capacities. The functional moieties present on their surface can be utilized to bind specific targeting moieties thereby achieving active targeting by the use of dendrimers. The other features of dendrimers include their biocompatible nature, robustness, and solubility in aqueous media, which calls for their internal use for humans. PPI- (polypropylene imine-), PEI- (polyethyleneimine-), and PAMAM-based dendrimers are mostly utilized biomedically. PAMAM is the most commonly used class of dendrimers having an alkyl diamine core along with tertiary branches. Polyester, polypeptide, triazine, and polyglycerol dendrimers are organic-based dendrimers that can be attached to the drug molecule to increase the efficacy of treatment. Hybrid and complex structures with other entities have been synthesized, and out of that, PEGylation is the most common technique to increase the blood circulation of dendrimers in blood and to avoid immune clearance [45].

PAMAM dendrimers have shown their potential in oral drug delivery because of their water solubility. As stated earlier, one marketed product out of that is Starburst® as it is characterized by its tree-like branching structure. Each series of branching is called generation, and each generation results in the increase of the dendrimer's structure, size, mass, and geometry. They are promising drug delivery systems due to the high degree of branches which can be terminated with a cationic amine (−NH_2_), neutral hydroxyl (−OH), or anionic carboxylic acid (−COOH) surface groups, which allow the conjugation of biological agents to a compact system. But the oral drug delivery by using dendrimers also faces some challenges which are due to their larger size and high molecular weight. Also, a small amount of lipophilicity is essential for the partition of drug molecules into the cell membrane. To get rid of these problems, some penetration enhancers are added to the formulation. Oral administration of proteins and enzymes had always been a big challenge for pharmaceutical industries due to enzyme degradation in gastrointestinal tract. Attempts had been made to decrease the protein degradation by formulation of a complex of the therapeutic entity with the dendrimers and administrating them with enzyme inhibitors [45].

Initial studies have confirmed that PAMAM dendrimers can bind to transcellular and paracellular routes and thus get penetrated to epithelial junctions which enhance their transport via paracellular routes [46].  Table 3 depicts the role of dendrimers as drug delivery agents.

### 5.2. Nasal Drug Delivery

Nasal drug delivery is an interesting alternative to the highly invasive parenteral route of drug delivery to achieve the high bioavailability of the drug. In this field also, PAMAM dendrimers have shown their potential to achieve nose to brain targeting of the drugs. Many groups or compounds can be attached to their surface like small interference RNAs (siRNAs) and arginine. The potential of dendrimers as mucoadhesive gels for intranasal delivery for the nose to brain targeting was further evaluated by Perez et al. [52]. They formulated dendriplexes by combining siRNA with PAMAM dendrimers and further formulated these particles into mucoadhesive gels with either 1% *w*/*v* chitosan or 0.25% carbopol 974P. Further in situ gelation was obtained by combining these with the thermosensitive polymer. The prepared gel was tested and found to be nontoxic at various concentrations.

### 5.3. Anticancer Drug Carriers

Studies using dendrimers utilized their unicellular structure for the noncovalent encapsulation of drugs [53]. DNA was complexed with PAMAM dendrimers for gene delivery to treat genetic disorders [54]. Similarly, hydrophobic drugs and dye molecules can be incorporated into dendrimers from the treatment as well as diagnostic aspects. The advantage of the unilamellar structure and rigid polymeric structure overruled the use of polymeric micelles in drug delivery as the bonds are covalently bounded here. However, this approach also suffers from the disadvantage of noncontrollable drug release as sometimes, harsh conditions are needed to get the drug released to the environment while sometimes, it becomes difficult to control the release of the drug. To get rid of this problem, a multivalent property of dendrimers was utilized, and multiple drugs can be covalently attached to the different groups present in the dendrimers. This covalent linkage of the drugs with the dendritic polymer may augment the pharmacological properties of the drugs.

Cisplatin was incorporated covalently in PAMAM dendrimers and has higher accumulation in solid tumors, lesser toxicity, and slower release as compared to the pure drug which was obtained for the same [54]. In this regard, Malik et al. [55] further formulated anticancer prodrug complexes with carboxylated terminated PAMAM dendrimers of cisplatin. They observed that the release mechanism for cisplatin from the dendrimer was hydrolysis, much higher maximum tolerated dose, higher survival period of tumor-bearing mice, and prolonged release of the drug for a much higher time. Further, they concluded that dendritic polymer can be administered orally, intramuscularly, parentally, subcutaneously, and topically to the animal with a malignant tumor and treated the tumor efficiently.

**Figure 3:**
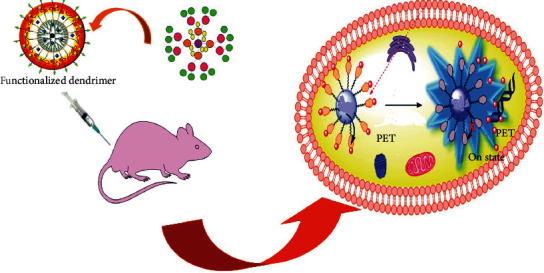
Dendrimers as drug delivery agents for the treatment of cancer.

Folate receptors are overexpressed in most of the cancer cells like ovary, breast cancer cells, kidney, and brain. So, folate ligands have been considered the most significant targeting moiety for cancer targeting. It also possesses good solubility and receptor binding properties and can be conjugated to a variety of conjugates including dendrimers, so folate-conjugated dendrimers are the focus of most researchers nowadays. Similarly, monoclonal antibodies can also be used for dendrimer-based cancer targeting as they can also selectively bind to tumor-associated antigens [56]. The descriptive diagram of anticancer drug carriers is presented in Figure 3.

### 5.4. Diagnostic Agents for Cancer

The ability of binding of the tumor-targeting antibody or any moiety and the anticancer drug conjugate to a single dendrimer provides a platform for treatment as well as diagnosis of cancer, but the safety of these molecules should be consistently studied before proceeding for these. Also, dendritic molecules can be conjugated with a variety of fluorescent molecules which can be extensively used to characterize the surface targeting, cell targeting, internalization, and drug biodistribution in the various organs. Various radioisotopes have been conjugated with the dendrimers, viz., 3H, 14C, 88Y, and III In [57–60]. With the help of these, chemical and physical properties of the dendrimers were adjusted so that they can favor biodistribution of the drugs; also, we can visualize the cancerous area with the help of radiolabeled isotopes. However, the method proposed here involves a postadministration dissection of the cancer tissues so that they can be investigated histologically. This technique has served as a stepping stone towards the minimum invasive or noninvasive clinical investigation procedures.

Although much research has been carried out exploring the use of dendrimers as a cancer diagnostic as well as treatment agent, still, their clinical use is in its infancy. However, their use in cancer therapy and diagnosis might be a valuable baby step because of their unique properties of accumulation or biodistribution inside the cancerous cells.

## 6. Future Prospective

The applications of dendrimers in drug delivery via various routes like oral, nasal, transdermal, and parental have been studied and well exploited nowadays. Their structural properties have been studied widely and found to be responsible for their unique characteristics like high payload and tissue accumulation [4, 61]. Further, they are found to have greater emphasis on gene delivery, boron neutron capture therapy, and magnetic resonance imaging (MRI) contrast agents [9]. Still, their applications in the medical and biomedical field need to reach the milestone. With improved synthesis, further understandings of their unique characteristics, and recognition of new applications, dendrimers will become promising candidates for further exploitation in drug discovery and clinical applications.

## 7. Conclusion

Dendrimers are the chemically distinguished entities with modifiable biological properties. They are known for their distinguished properties which make them hopeful candidates for the number of applications. Although they were studied for the past two decades, their synthesis requires multistep chemical reactions. Several studies concluded that dendrimers can be a breakthrough success for the treatment and diagnosis of cancer. Dendrimers can also work as a useful tool for the optimization of drug delivery systems. Moreover, the structural properties of dendrimers like their shape, structure, size, branching, functionality, void space, and density made them an ideal candidate for drug delivery by various routes.

## Figures and Tables

**Table 1:** Properties of dendrimers [3].

S. No.	Properties	Dendrimer

1	Structure and shape	Compact and spherical/globular
2	Size	Range of 1-100 nm
3	Architecture	Regular
4	Structural control	Very high
5	Synthesis	Stepwise growth
6	Crystallinity	Noncrystalline, amorphous materials, low glass temperatures
7	Reactivity	High
8	Aqueous solubility	High
9	Nonpolar solubility	High
10	Viscosity	Nonlinear relationship with molecular weight
11	Ionic conductivity	High
12	Compressibility	Low
13	Polydispersity	Monodisperse/narrow polydispersity index
14	Compressibility	Low

**Table 2:** Dendrimers as anticancer drug carriers.

S. No.	Drug	Mechanism of interaction	Result	Reference

1	5-Fluorouracil	Chemically conjugated system	Solubility enhancement of 5-fluorouracil	[39]
2	Cisplatin	Covalent bonding	Reduced cytotoxicity and improved entrapment efficiency	[40]
3	Doxorubicin	Encapsulation	Solubility enhancement	[41]
4	Methotrexate	Encapsulation	Improved bioavailability	[42]
5	Paclitaxel	Encapsulation	Solubility enhancement	[43]
6	Cisplatin	Encapsulation	Reduced toxicity	[43]

**Table 3:** Description of research on dendrimers.

S. No.	Description	Outline	Reference

1	Synthesized prodrug by binding propranolol with lauroyl 3G PAMAM dendrimer and determined the effect of propranolol on adenocarcinoma Caco-2 cells.	Apical to basolateral (A-B) permeability coefficient (Papp) of propranolol was increased, but B-A Papp was decreased. Further observed that A-B Papp was decreased in the presence of colchicine.	[47]
2	Prolonged delivery of ketoprofen using PAMAM dendrimers was attempted by in vitro studies and in vivo studies.	—	[48]
3	Citric acid-polyethylene glycol-citric acid copolymers were prepared, and hydrophobic drugs like mefenamic acid and pyridine were instilled into the guest cavity.	The hydrophobic molecules, when entrapped into hydrophilic cavities, became soluble in aqueous solution.	[49]
4	Aqueous formulation of indomethacin was prepared with increasing concentration of dendrimers to achieve the transdermal drug delivery.	The steady-state flux of the drug increases significantly and was highest with –NH_2_ dendrimer at 0.2% *w*/*v* concentration. Also, in vivo steady-state flux was reached after 5 hrs, and the highest steady-state concentration values were found with –NH_2_ and –OH dendrimers.	[15]
5	This investigation was carried out to evaluate the potential of PAMAM dendrimers with three different functional group carriers so that they can be used as drug carriers. Drug dendrimer complexes were evaluated for solubility, stability, and in vitro release studies.	The PEGylated polymers showed maximum solubility enhancement followed by amine and hydroxyl dendrimers.	[50]
6	The gel formulations of 1%, 3%, and 5% (wt/wt) of SPL7013 (dendrimer having antiviral activity) were prepared and evaluated for the vaginal and rectal safety profile.	The vaginal safety profile of the 1% & 3% gel containing polymer were the same, but they were superior to that of 5%. The rectal safety profile of 3% gel was also good so they can be used for internal use.	[51]
